# Validation of the Romanian Version of the 6-Item Carpal Tunnel Syndrome Symptoms Scale and Palmar Pain Scale Questionnaire

**DOI:** 10.3390/jcm14093059

**Published:** 2025-04-29

**Authors:** Daniel-Corneliu Leucuța, Nicu Catalin Draghici, Maria Geanina Balea, Roxana Toader, Hanna Maria Dragos, Livia Livinț Popa, Dafin Fior Mureșanu

**Affiliations:** 1 Department of Medical Informatics and Biostatistics, “Iuliu Hatieganu” University of Medicine and Pharmacy, 400349 Cluj-Napoca, Romania; dleucuta@umfcluj.ro; 2 Department of Clinical Neurosciences, “Iuliu Hatieganu” University of Medicine and Pharmacy, 400012 Cluj-Napoca, Romania; hanna_dragos2006@yahoo.com (H.M.D.); livia.popa@umfcluj.ro (L.L.P.); dafinm@ssnn.ro (D.F.M.); 3 IMOGEN Institute, Centre of Advanced Research Studies, 400347 Cluj-Napoca, Romania; 4 RoNeuro Institute, Centre for Neurological Research and Diagnostic, 400364 Cluj-Napoca, Romania; maria.balea@ssnn.ro (M.G.B.); roxana.ailoaei@ssnn.ro (R.T.)

**Keywords:** carpal tunnel syndrome, CTS-6, validation, Romanian, symptom scale, psychometric properties, electrophysiology

## Abstract

**Background/Objectives:** Carpal tunnel syndrome (CTS) is a common neuropathy that significantly impacts patients’ quality of life and incurs substantial healthcare costs. The 6-item Carpal Tunnel Syndrome Symptom Scale and the Palmar Pain Scale (PPS) are concise, reliable tools widely used to assess CTS symptom severity. Our study aimed to translate, culturally adapt, and validate the Romanian version of the 6-item CTS Symptoms Scale in a sample of Romanian-speaking patients. **Methods:** This cross-sectional study involved 118 wrists from 59 Romanian-speaking patients. Each participant completed the CTS-6 scale and nerve conduction studies were carried out on their wrists. The CTS-6 scale was translated into Romanian using a forward–backward translation process. The psychometric properties of the Romanian CTS-6 were assessed, including internal consistency and construct validity (criterion validity, convergent, and divergent validity). **Results:** The Romanian CTS-6 and PPS demonstrated high internal consistency (Cronbach’s alpha = 0.93, respectively, 0.92, and strong item-total correlations). Factor analysis confirmed their unidimensional structure, with factor loadings ranging from 0.80 to 0.90. The CTS-6 scores showed moderate correlations with electrophysiological parameters, supporting criterion validity. Divergent validity was shown too. **Conclusions:** The Romanian version of the 6-item CTS Symptoms Scale and PPS is a valid tool for assessing CTS symptom severity in Romanian-speaking populations.

## 1. Introduction

Carpal tunnel syndrome (CTS) is a musculoskeletal condition caused by the compression of the median nerve as it travels through the carpal tunnel in the wrist. CTS is characterized by symptoms of pain, tingling, and numbness in the hands and fingers [1]. CTS has important but varying prevalence rates across different populations and regions, largely influenced by factors such as occupation, gender, and health conditions [1,2,3].

CTS is diagnosed through a combination of clinical evaluation, physical tests, and confirmatory diagnostic tools like nerve conduction studies (NCSs) and imaging [4]. The clinical evaluation is still important for differential diagnosis and discriminating from other possible neuropathic conditions of the hand. Several diagnostic questionnaires have been developed and validated to aid in the diagnosis and assessment of carpal tunnel syndrome. One of the most widely used specific tools is the Boston Carpal Tunnel Questionnaire (BCTQ), which evaluates symptom severity and functional disability [5]. The BCTQ was the first questionnaire developed for this aim, is comprehensive, and is used extensively in research. Another wildly used tool is the Six-Item Carpal Tunnel Syndrome (CTS-6) scale, which was developed by Atroshi et al. in 2011 as a shorter version of the original BCTQ [6]. This reduction aimed to maintain accuracy while improving the speed of administration without sacrificing the scale’s reliability or validity. Indeed, the CTS-6 has demonstrated high internal consistency, reliability, and validity [7,8,9]. Atroshi et al., 2011, also developed a two-item Palmar Pain Scale (PPS) as a measure of pain after surgical intervention in CTS [6].

There is no translated and validated Romanian CTS-6 scale or PPS. Language and cultural differences can affect how participants respond to questionnaires and influence their validity; thus, culturally adapted questionnaires translated to a different language are a necessity. Therefore, this study aimed to translate, culturally adapt, and assess the construct validity, reliability, and convergent and divergent validity of the CTS-6 scale in a Romanian cohort.

## 2. Materials and Methods

### 2.1. Study Design and Setting

A cross-sectional observational study was designed to translate, culturally adapt, and validate the 6-item Carpal Tunnel Syndrome (CTS-6) scale into Romanian. The study was conducted at RoNeuro, a specialized neurological center, and the Imogen Institute in Cluj-Napoca, Romania, between October 2023 and May 2024.

### 2.2. Participants

Eligible participants included adults (aged 18 and above), Romanian-speaking participants with sufficient literacy to complete self-reported questionnaires, and those who had not previously undergone surgical treatment for CTS and were referred for CTS based on clinical symptomatology. Individuals with major psychiatric conditions or an inability to understand the Romanian language were excluded from the study.

### 2.3. Ethical Approval

The study was approved by the Ethics Committee of the Iuliu Hațieganu University of Medicine and Pharmacy, Cluj-Napoca, Romania, on 17 February 2024, document number 20. Written informed consent was obtained from all participants before inclusion.

### 2.4. Translation and Cultural Adaptation

The CTS-6 scale and PPS were translated into Romanian following established guidelines for cross-cultural adaptation [10]. Three independent translators, all native Romanian speakers fluent in English and unfamiliar with the questionnaire, produced two forward translations. A professional linguist (teacher of the Romanian language) also amended the text to ensure correct grammar and readability. A consensus version was created by reconciling the two independent translations and the Romanian correction through discussions with neurologist specialists. The reconciled version was back-translated into English by an independent translator who was a native English speaker but also fluent in Romanian. The back-translator was blinded to the original English version of the CTS-6 and PPS. An expert panel consisting of clinicians and methodologists reviewed all versions to resolve any discrepancies and ensure semantic, idiomatic, and conceptual equivalence. Modifications were made as needed to ensure cultural relevance and comprehension. The final version can be found in Appendix A.

### 2.5. Data Collection

We collected data on clinical, demographic, and electrophysiological measurements. Demographic information such as age, sex, height, weight, place of residence, education, and hand dominance was recorded. Clinical data included the duration of CTS symptoms and a history of active repetitive movements. Additionally, all participants completed the Romanian-translated CTS-6 and PPS. All participants were assessed using NCSs on both wrists.

### 2.6. Psychometric Evaluation

The psychometric evaluation of the Romanian CTS-6 consisted of an internal consistency assessment and construct validity assessment (by convergent, criterion, and discriminant validity).

### 2.7. 6-Item CTS and PPS

The 6-CTS is a concise self-assessment tool designed to measure the severity of CTS symptoms [6]. The 6-CTS consists of six symptom-focused questions. Four of these items evaluate the severity of specific symptoms over a 24 h period during the past two weeks: (1) pain at night, (2) pain during the daytime, (3) numbness or tingling at night, and (4) numbness or tingling during the daytime. These are scored on a 5-point Likert scale ranging from “None” (1) to “Very severe” (5). The remaining two items assess how often these symptoms wake the patient at night—specifically pain, numbness, or tingling, respectively—with response options ranging from “Never” (1) to “More than 5 times” (5). The total score is calculated by summing the responses for all six items, ranging from 6 to 30, with higher scores reflecting greater symptom severity. This questionnaire’s short format is useful in clinical settings where the assessment time is limited. The main use is in measuring symptom severity and monitoring treatment effectiveness. Although short, the 6-CTS has very good psychometric properties, with high internal consistency, reliability, and validity [7,8,9]. The PPS is a measure of pain after the surgical intervention for CTS. It comprises two items: The first assesses the intensity of pain or tenderness, with options ranging from “None” to “Very severe” on a 6-point Likert scale. The second item evaluates the functional impact of this discomfort on the patient’s daily activities, with responses from “Not at all” to “Extremely”. The PPS adds value in post-intervention monitoring, ensuring that pain-related complications or limitations are not overlooked.

### 2.8. Nerve Conduction Studies

Electrophysiological testing was conducted for each participant to assess the presence of CTS and assess median nerve function. Two trained neurophysiologists with 10 years of experience in electroneurography performed all the measurements. Measurements included the Median Compound Muscle Action Potential (CMAP) amplitude in millivolts (mV) and the Median CMAP Latency in milliseconds (ms). Sensory function was assessed using the Sensory Nerve Action Potential (SNAP) amplitude in microvolts (µV), and Antidromic Median Nerve NCS velocity for Digit II in meters per second (m/s). To rule out an ulnar nerve neuropathy at the elbow or demyelinating chronic inflammatory polyradiculoneuritis, similar measurements were taken for the ulnar nerve, including the Ulnar Compound Muscle Action Potential amplitude (mV), Ulnar CMAP Latency (ms), Antidromic Ulnar Sensory Nerve Action Potential amplitude (µV), and Antidromic Ulnar Nerve Conduction Study velocity for Digit V (m/s). CTS electrodiagnosis was performed following the recommendations of the American Association of Neuromuscular & Electrodiagnostic Medicine [11]. The diagnostic criteria for CTS were an antidromic median sensory nerve conduction velocity < 40 m/s and/or a median motor nerve distal latency ≥ 4.2.

### 2.9. Statistical Analyses

An a priori sample size calculation was performed. Approximately 10 participants per questionnaire item were aimed for to ensure sufficient statistical power for validation analyses based on recommendations for validation studies [12]. Since both wrists were assessed for each participant, the sample size was almost double the suggested threshold.

The internal consistency of a questionnaire can be measured with Cronbach’s alpha and Guttman’s Lambda to see the relation between the items (questions) and whether they represent the latent construct one wants to measure. Cronbach’s alpha is the most used internal consistency measure and is easier to compute, while Guttman’s Lambda is more robust in terms of heterogenous items. Cronbach’s alpha coefficient for reliability was computed with 95% Duhachek confidence intervals [13]. According to Nunnally (1994), Cronbach’s alpha values over 0.8 are considered appropriate [14]. Also, Guttman’s Lambda was calculated for the same purpose. Furthermore, the average inter-item correlation was computed. To check the impact of each item on the questionnaire, each item was dropped, and the previous statistics were computed for the remaining items. Exploratory factor analysis (EFA) was performed, applying principal axis factoring for factor extraction and varimax rotation to the extracted factors. Bartlett’s test of sphericity, as well as the overall and individual Kaiser–Meyer–Olkin (KMO) measure of sampling adequacy, were used checked as assumptions before performing the EFA. Eigenvalues and a scree plot were used to identify how many factors might be sufficient for capturing the major variance component of the dataset. Construct validity assesses how well a tool measures the concept it is supposed to measure. This type of validity can be broken down into two subtypes: convergent and discriminant validity. Convergent validity is important when the tool being tested shows high correlations with another tool that is designed to measure the same or similar concepts. On the other hand, discriminant (or divergent) validity occurs when tools designed to measure different concepts exhibit little to no correlation. The convergent validity was analyzed with Spearman correlations between the 6-item CTS Symptoms Scale and electrophysiological severity as measured by the Median CMAP Latency (ms) and Antidromic Median NCS (DII) (nerve conduction studies are the best available measure of impaired nerve function [4]) (which, in this case, is also a concurrent validity criterion), while divergent validity was analyzed by carrying out correlations with age and body mass index [15]. The correlation coefficient interpretation in absolute value was as follows: [0–0.25)—no or weak; [0.25–0.50)—moderate; [0.50–0.75)—good; [0.75–1]—very good [16]. Since several correlation coefficients were calculated, a Holm adjustment for the p-value was performed. The psych R package version 2.4.6.26 was used for psychometric analyses [17]. For all analyses, the R environment for statistical computing and graphics version 4.3.2. was used [18].

## 3. Results

The sample consisted of 118 wrists from 59 participants with a mean age of 57.1 years (standard deviation of 14.4, ranging from 19 to 86 years old (Table 1)). Most of them were female and were right-handed. The average body mass index was 29.6 kg/m^2^, indicating an overweight to obese majority. Most participants resided in urban areas and had at least a high school education (81.4%). Over half acknowledged repetitive hand movements. The symptom duration was variable, with a median of 18 months for the right hand and 12 months for the left. A small subset had previously undergone surgery for CTS.

### 3.1. Exploratory Factor Analysis

Bartlett’s test of sphericity gave a *p*-value less than 0.001, suggesting that the items are correlated enough to allow for the use of factor analysis. The overall Kaiser–Meyer–Olkin (KMO) measure of sampling adequacy was 0.76 (with above 70 being considered good), indicating a high proportion of variance among items, which might be a common variance. This result suggests the appropriateness of factor analysis. Furthermore, all individual KMO values were above 0.70, showing that each item has sufficient variation in common with the other items for factor analysis to be carried out successfully.

The scree plot suggests that factors beyond the very first one do not significantly enhance explanatory power and may be omitted from subsequent analyses. The first factor has an eigenvalue of 4.23, so it explains 4.23/6 items × 100 = 70% of the variance in all items. All the other factors have eigenvalues below one, suggesting that the items point to one single latent factor.

The factor loadings (Table 2) are consistently high across all items, indicating a strong relationship with the underlying factor. The uniqueness values, although differing among items, suggest that most of the variance of each item is accounted for by the factor. The lowest uniqueness was observed for the first item, suggesting that most of its variance is accounted for by the factor, while the highest uniqueness was observed for the last item, with the least explained variance by the common factor. Overall, the one-factor model is effective in capturing the common underlying construct.

### 3.2. Reliability

#### 3.2.1. Item Statistics

The CTS-6 questionnaire shows excellent reliability—Cronbach’s alpha = 0.93—with a precise result, having a narrow confidence interval (95% CI 0.92–0.95). Further confirmation of this result is made by the similar value of Guttman’s Lambda of 0.95. The average inter-item correlation was 0.7, supporting the internal consistency of the questionnaire (Table 2). All items contributed positively to the reliability of the questionnaire. Dropping any item did not significantly increase Cronbach’s alpha or other reliability statistics.

The PPS showed excellent reliability too—with a Cronbach’s alpha of 0.92 (95% CI 0.91–0.94) and a Guttman’s Lambda of 0.95. The internal consistency is supported by a very good inter-item correlation of 0.95. All items contributed positively to the reliability of the questionnaire. Dropping any item did not significantly increase Cronbach’s alpha or other reliability statistics. However, there was a low variability in response; most respondents chose the first option.

#### 3.2.2. Validity

Convergent validity was sustained by significant adjusted correlations with electrophysiological measurements (Table 3). These correlations also sustain concurrent criterion validity. There was a moderate inverse proportional correlation with Antidromic Median NCS digit II and a moderate direct proportional correlation with Median CMAP Latency. Divergent validity was suggested by the absence of significant adjusted correlations with age and body mass index.

## 4. Discussion

This study aimed to translate, culturally adapt, and validate the CTS-6 for a Romanian-speaking population. Our findings support the reliability and validity of the Romanian version of the CTS-6. The translated scale showed high internal consistency, strong item–total correlations, and robust factor loadings, confirming its one-dimensionality and its alignment with the original construct of CTS symptom severity. Furthermore, the study showed the convergent validity (and concurrent criterion validity) of the 6-CTS through significant correlations with electrophysiological measurements, as well as its divergent validity.

There are studies that limit themselves to the translation of CTS-6 in different languages without making sure that their psychometric properties are retained [19]. Our study went further to assess these properties, too.

In our study, we found good internal consistency for the CTS-6 questionnaire (0.93). Our result is similar to results from other studies that translated and validated the CTS-6 questionnaire. Rosales et al. (2016) created a Spanish version of the CTS-6, and their Cronbach alpha was 0.81, suggesting a good internal consistency [9]. Belka et al., 2021, adapted the questionnaire to Polish and identified a 0.81. Cronbach alpha value [20]. The alpha value for the Norwegian version of the CTS-6 questionnaire by Schulze et al., 2021, was 0.82 [7]. A similar value was found for the Turkish version of the CTS-6 (0.829) by Salbas et al. 2023 [8]. These results show that the questionnaire maintains its reliability across varied cultural contexts.

The value of Cronbach’s alpha was high, being appropriate for sustaining its reliability, as following Nunnally’s [14] interpretation [14]. The obtained value is not too high either, with values over 0.95 being considered too high by George and Mallery (2003) [21]. Streiner (2003) [22] indicates that values over 0.90 indicate some degree of redundancy between the items of the questionnaire, but this does not diminish the value of the instrument.

The original CTS-6 had a one-factor structure. We also found arguments in our study to sustain the same one-dimensionality during the exploratory factor analysis. The same result was observed for the Turkish version of the CTS-6 [8].

The reproducibility of the original CTS-6 was very high (0.95) [23], as was the reproducibility of the translated versions ranging from 0.85 to 0.87 [7,8,9]. Although our study did not assess this characteristic, it is likely that similar values would apply to it too.

Few other CTS-6 translations performed nerve conduction velocity to assess criterion validity as we did in our study. NCS is an objective measure that was found to be correlated with the Romanian CTS-6 version.

### 4.1. Limitations

There are several limitations to this research. Although the sample size was more than enough for the validation, the generalizability of the results could be helped by a larger sample size. The study could have been better if it had included patients from more diverse healthcare settings. Due to the cross-sectional design, we were unable to evaluate the scale’s response to changes over time (e.g., after therapy). Future studies should examine how well the Romanian CTS-6 assesses the evolution of symptoms and the results of medical interventions over time.

### 4.2. Strengths

This study has several strengths. Psychometric analysis is the key strength of the study, as it guarantees that the tools are accurate, reliable, and clinically relevant. It involved evaluating the consistency of response, and the validity and accuracy of the tools for measuring CTS symptoms, as well as postoperative pain. This way, we validated their usage as standardized, evidence-based measurements for clinical practice and research. The translation followed a rigorous process, ensuring cultural and conceptual alignment with the original CTS-6. Incorporating electrophysiological measurements added to the clinical relevance by demonstrating criterion validity. This is the first translation and validation of the CTS-6 and PPS for the Romanian population.

## 5. Conclusions

The Romanian versions of the 6-item CTS Symptoms Scale and Palmar Pain Scale questionnaire have high internal consistency and present valid tools for measuring symptom severity in CTS patients. Their psychometric properties are similar to those of the original scales and their translations, making them the first choice for assessing symptoms and treatment efficacy in Romanian-speaking populations within both clinical care and research on CTS.

## Figures and Tables

**Table 1:** Patient characteristics.

Characteristic	N = 59
Age (years), mean (SD) [range]	57.1 (14.4) [19–86]
Female, n (%)	41 (69.5)
Body mass index (kg/m^2^), mean (SD) [range]	29.6 (5.8) [19.2–50.2]
Handedness, n (%)	
Right	53 (89.8)
Left	3 (5.1)
Ambidextrous	3 (5.1)
Urban, n (%)	37 (62.7)
Education, n (%)	
Primary school	11 (18.6)
High school	33 (55.9)
University	15 (25.4)
Active repetitive movements, n (%)	36 (61.0)
Symptom duration (months), median (IQR)	
Right	18 (2–36)
Left	12 (3–24)
Surgery for carpal tunnel syndrome, n (%)	4 (6.8)

BMI, body mass index; SD, standard deviation; IQR, interquartile range.

**Table 2:** Results of the Six-Item Carpal Tunnel Syndrome (CTS-6) scale and Palmar Pain Scale (PPS) item description, item-total correlation analysis, factor loadings, and uniqueness.

Question	Mean (SD)	Corrected Item–Total Correlations	Factor Loadings	Uniqueness
CTS-6 items				
How severe are the following symptoms in your hand?				
Pain at night	2.1 (1.3)	0.90	0.90	0.20
Pain during daytime	2.1 (1.1)	0.83	0.81	0.34
Numbness or tingling at night	2.7 (1.2)	0.88	0.87	0.24
Numbness or tingling during daytime	2.5 (1.0)	0.84	0.83	0.30
How often did the following symptoms in your hand wake you up at night?				
Pain	2.0 (1.1)	0.82	0.82	0.33
Numbness or tingling	2.2 (1.0)	0.80	0.80	0.37
Palmar pain scale				
How much pain or tenderness do you have in the surgical scar or palm?				
	1 (0.21)	0.95		
How much does the pain or tenderness in the surgical scar or palm limit your activities?				
	1 (0.13)	0.95		

SD, standard deviation.

**Table 3:** Spearman correlation coefficients between the 6-item Carpal Tunnel Syndrome (CTS) Symptoms Scale and electrophysiological measurements, age, and body mass index (BMI).

Characteristics	6-Item CTS Symptoms Scale	Antidromic Median NCS Digit II (m/s)	Median CMAP Latency (ms)	Age	BMI (kg/m^2^)
6-item CTS Symptoms Scale	1				
Antidromic Median NCS digit II (m/s)	−0.39 (0.001)	1			
Median CMAP Latency (ms)	0.29 (0.009)	−0.66 (<0.001)	1		
Age	0.08 (1)	−0.33 (0.002)	0.134 (0.649)	1	
BMI (kg/m^2^)	0.22 (0.083)	−0.03 (1)	−0.0001 (1)	0.14 (0.649)	1

NCS, nerve conductivity study; CMAP, compound muscle action potential; BMI, body mass index; Holm adjusted *p*-values are shown in brackets.

## Data Availability

Dataset available on request from the authors.

## Abbreviations

The following abbreviations are used in this manuscript:

CTS	Carpal Tunnel Syndrome
CTS-6	6-item CTS Symptoms Scale
PPS	Palmar Pain Scale
NCS	Nerve Conduction Studies
BCTQ	Boston Carpal Tunnel Questionnaire
CMAP	Median Compound Muscle Action Potential
SNAP	Sensory Nerve Action Potential
EFA	Exploratory factor analysis
KMO	Kaiser–Meyer–Olkin
BMI	Body Mass Index
SD	Standard deviation
IQR	Interquartile range

## Appendix A

Scala celor 6 simptome în Sindromul de tunel carpian

Următoarele întrebări se referă la simptomele dumneavoastră pentru o perioadă tipică de 24 de ore în cursul ultimelor 2 săptămâni.

(Vă rugăm să marcați câte un răspuns pentru fiecare simptom.)

* *

Cât de severe sunt următoarele simptome de la nivelul mâinii?

	**Deloc**	**Ușoare**	**Moderate**	**Severe**	**Foarte Severe**
Durere în cursul nopții					
Durere în timpul zilei					
Amorțeală/ furnicături în timpul noptii					
Amorțeală/furnicături în timpul zilei					

Cât de des v-au trezit din somn următoarele simptome de la nivelul mâinii?

	**Niciodată**	**o dată**	**de 2-3 ori**	**de 4-5 ori**	**Peste 5 ori/noapte**
Durere					
Amorțeală/ furnicături					

Scala durerii palmare în sindromul de tunel carpian

Care este gradul de durere sau sensibilitate pe care îl resimțiți la nivelul cicatricei chirurgicale sau în palmă?

☐ Deloc ☐ Foarte ușor ☐ Ușor ☐ Moderat ☐ Sever ☐ Foarte sever

* *

Cât de mult vă limitează durerea/sensibilitatea din palmă sau de la nivelul cicatricei chirurgicale?

☐ Deloc ☐ Puțin ☐ Moderat ☐ Destul de mult ☐ Extrem de mult
